# Overcoming the mechanisms of primary and acquired resistance to new generation hormonal therapies in advanced prostate cancer: focus on androgen receptor independent pathways

**DOI:** 10.20517/cdr.2020.42

**Published:** 2020-09-12

**Authors:** Maristella Bungaro, Consuelo Buttigliero, Marcello Tucci

**Affiliations:** ^1^Medical Oncology, University of Turin, San Luigi Gonzaga Hospital, Orbassano, Turin 10043, Italy.; ^2^Medical Oncology, Cardinal Massaia Hospital, Asti 14100, Italy.

**Keywords:** castration-resistant prostate cancer, androgen receptor signalling, hormonal treatment, enzalutamide, abiraterone, resistance mechanisms

## Abstract

In recent years, many therapeutic advances have been made in the management of castration-resistant prostate cancer, with the development and approval of many new drugs. The androgen receptor (AR) is the main driver in prostate cancer growth and progression and the most effective therapeutic agents are still directed against this pathway. Among these, new generation hormonal agents (NHA) including enzalutamide, abiraterone acetate, apalutamide, and darolutamide have shown to improve overall survival and quality of life of prostate cancer patients. Unfortunately, despite the demonstrated benefit, not all patients respond to treatment and almost all are destined to develop a resistant phenotype. Although the resistance mechanisms are not fully understood, the most studied ones include the activation of both dependent and independent AR signalling pathways. Recent findings about multiple growth-promoting and survival pathways in advanced prostate cancer suggest the presence of alternative mechanisms involved in disease progression, and an interplay between these pathways and AR signalling. In this review we discuss the possible mechanisms of primary and acquired resistance to NHA with a focus on AR independent pathways.

## Introduction

Prostate cancer (PC) is the most frequent cancer in men and accounts for almost 1 in 5 new diagnoses. Although in recent years there has been a decrease in mortality due to earlier detection and advances in treatment, PC is still the second leading cause of cancer death among men in the USA^[1]^. At the time of diagnosis, most patients have a localized disease treatable with surgery or radiotherapy with curative intent. About 5%-10% of PC is diagnosed at an advanced stage. While 5-year survival is close to 100% for localized and locally advanced disease, it is nearly 30% in metastatic patients^[2]^. Androgen deprivation therapy (ADT), both surgical and biochemical, is the standard of care in metastatic castration-sensitive prostate cancer (mCSPC), owing to the driving role of androgen receptor (AR) in the growth of this tumour. Recent findings demonstrated that, in patients with high volume de novo mCSPC, the addition of docetaxel to ADT results in significantly improved outcomes when compared with ADT alone^[3,4]^. Similar results have been recently presented for the combination of new generation hormonal treatments and ADT in high risk mCSPC^[5-7]^. Despite the brilliant response rates (approximately 80%-90%), almost all patients experience disease progression within 18-36 months from the start of ADT, moving to the last phase of disease, castration-resistant prostate cancer (CRPC)^[8]^. Recently, many therapeutic advances have been made in metastatic CRPC (mCRPC), with the development and approval of new generation orally available hormonal agents: abiraterone, a strong CYP17A1 inhibitor blocking androgens biosynthesis, and enzalutamide, a potent AR antagonist. The phase III trials COU-AA-301^[9]^ and AFFIRM^[10]^ demonstrated an improvement in overall survival (OS) in patients treated respectively with abiraterone and enzalutamide in post-docetaxel setting [Table 1]. In the COU-AA-302 study, treatment with abiraterone acetate prolonged OS compared with prednisone alone in asymptomatic and mildly symptomatic chemotherapy-naïve patients^[11]^ [Table 1]. In a population of patients with similar characteristics, the PREVAIL study showed that enzalutamide significantly decreases the risk of death compared to placebo^[12]^ [Table 1]. Unfortunately, after an initial response PC cells become resistant even to NHA, leading to the progression of disease. Other drugs were consequently tested to manage the resistance induced by abiraterone and enzalutamide. Apalutamide is an oral irreversible AR antagonist that prevents AR nuclear translocation. In the phase III SPARTAN trial, apalutamide, in addition to ADT, was demonstrated to reduce the risk of metastasis or death by 72% and to prolong median metastasis-free survival (MFS) in patients with high-risk, non-metastatic CRPC^[13]^ [Table 1]. Apalutamide was approved in 2018 for the treatment of non-metastastic CRPC patients. Efficacy in men with mCSPC was demonstrated in the phase III TITAN trial^[7]^ [Table 1]. In this study, apalutamide in addition to ADT demonstrated a significant improvement, compared to ADT alone, in both major efficacy outcomes of OS and radiographic progression-free survival (rPFS)^[7]^. In September 2019, the Food and Drug Administration (FDA) approved apalutamide for mCSPC patients. Darolutamide is a next generation AR antagonist, with higher affinity for AR than enzalutamide. Approval of darolutamide for the treatment of non-metastatic CRPC was based on the benefit in MFS reported in the phase III ARAMIS study^[14]^ [Table 1]. Enzalutamide showed overlapping results in the PROSPER trial^[15]^ [Table 1]. There are no direct comparative studies between NHA drugs. The clinical decision of which of the currently available agents to use is currently based on the toxicity profile of the drugs and on the comorbidities of the individual patient being treated. Most of the mechanisms responsible for disease progression in CRPC relates to AR signalling maintenance^[16]^. Recent studies on growth-promoting and survival pathways in advanced PC suggest the crucial role of alternative AR-independent mechanisms in disease progression and an interplay between these pathways and AR signaling^[17]^. In this review we discuss the possible mechanisms of primary and acquired resistance to NHA with a specific focus on AR independent pathways.

**Table 1:** Outcomes and toxicities of the pivotal NHA studies

Study name	Trial registration	Intervention/treatment	Outcome (primary endpoint)	Adverse events
COU-AA-301	NCT00638690	Abiraterone Acetate *vs*. Placebo in mCRPC previously treated with Docetaxel	OS 14.8 *vs*. 10.9 mo (HR = 0.65; 95%CI: 0.54-0.77; *p* < 0.001)	Fluid retention Hypertension Hypokalemia
AFFIRM	NCT00974311	Enzalutamide *vs*. Placebo in mCRPC previously treated with Docetaxel	OS 18.4 *vs*. 13.6 mo (HR = 0.63; 95%CI: 0.53-0.75; *p* < 0.001)	Fatigue Diarrhea Hot flushes
COU-AA-302	NCT00887198	Abiraterone Acetate *vs*. Placebo in Docetaxel-naive mCRPC	OS 34.7 *vs*. 30.3 mo (HR = 0.81; 95%CI: 0.70-0.93; *p* = 0.0033) rPFS 16.5 *vs*. 8.2 mo (HR = 0.52; 95%CI: 0.45-0.61; *p* < 0.0001)	Fluid retention Hypertension Hypokalemia
PREVAIL	NCT01212991	Enzalutamide *vs*. Placebo in Docetaxel-naive mCRPC	OS (est) 32.4 *vs*. 30.2 mo (HR = 0.71; 95%CI: 0.60-0.84; *p* < 0.001) 12 mo rPFS 65% *vs*. 14% (HR = 0.19; 95%CI: 0.15-0.23; *p* < 0.001)	Fatigue Hypertension
SPARTAN	NCT01946204	Apalutamide *vs*. Placebo in non metastatic CRPC	MFS 40.5 *vs*. 16.2 mo (HR = 0.28; 95%CI: 0.23-0.35; *p* < 0.001)	Rash Hypothyroidism Bone fracture
TITAN	NCT02489318	Apalutamide *vs*. Placebo in mCSPC	24 mo OS 84.2% *vs*. 73.5% (HR = 0.67; 95%CI: 0.51-0.89; *p* = 0.005) 24 mo rPFS 68.2% *vs*. 47.5% (HR = 0.48; 95%CI: 0.39-0.60; *p* < 0.001)	Rash Hot flushes Fatigue
ARAMIS	NCT02200614	Darolutamide *vs*. Placebo in non metastatic CRPC	MFS 40.4 *vs*. 18.4 mo (HR = 0.41; 95%CI: 0.34-0.50; *p* < 0.001)	Fatigue
PROSPER	NCT02003924	Enzalutamide *vs*. Placebo in non metastatic CRPC	MFS 36.6 *vs*. 14.7 mo (HR = 0.29; 95%CI: 0.24-0.35; *p* < 0.001)	Fatigue Hot flushes Hypertension

NHA: new generation hormonal agents; mCRPC: metastatic castration resistant prostate cancer, OS: overall survival; mo: months; HR: hazard ratio; CI: confidence interval; rPFS: radiographic progression free survival; est: estimated; MFS: metastasis free survival; mCSPC: metastatic Castration sensitive prostate cancer

## Androgen receptor structure and function

Androgens are sex hormones required for the development of the male reproductive system and secondary sexual characteristics. Testosterone (T) and 5-a-dihydrotestosterone (DHT) are mostly produced in the testicles in the adult male. No more than 10% of their synthesis occurs in the adrenal glands.

Testosterone and DHT act via the androgen receptor (AR), a ligand-dependent nuclear transcription factor, encoded by a gene located on chromosome Xq11-12. The receptor structure consists of three main functional domains: the N-terminal transcriptional regulation domain, the DNA binding domain and the ligand binding domain (LBD). In the absence of ligand, the AR is cytoplasmic, linked to heat-shock and other chaperone proteins. When androgens bind AR, they induce a conformational change resulting in dissociation of chaperone proteins and exposure of the nuclear localization signal, responsible for AR import into the nucleus. The androgen/AR complex translocates to the nucleus where it dimerizes and binds the androgen response elements to modulate gene transcription^[18]^. The transcriptional activity and the interaction with other pathways involved in cellular signalling is modulated by several coactivator and suppressor proteins^[19]^. The androgen/AR complex can also act through non-DNA binding-dependent pathways. Activation of second messenger signalling pathways have been identified in several cell lines^[20]^. There is evidence suggesting that some of the non-DNA binding-dependent actions of androgens are mediated via the activation of membrane-bound protein receptors, starting intracellular signalling cascades even in the presence of low levels of androgens^[21]^. The AR promotes physiological epithelial differentiation, but, in PC, abnormal AR activation can dysregulate the expression of genes involved in the control of proliferation and survival of tumour cells^[22]^.

## The adaptive response to conventional androgen deprivation therapy

In recent years there has been a significant increase in the understanding of prostate cancer biology. It is currently known that the shift from CSPC to CRPC is not necessarily due to androgen deprivation therapy (ADT) resistance, but it is caused by cell adaptation to a microenvironment with low androgens levels. In fact, despite the significant T levels decrease induced by ADT, CRPC remains driven by AR signaling^[23]^; PC cells are able to synthesize androgens and modify AR, enabling it to activate even in the presence of low levels of androgens. The mechanisms underlying this phenomenon are associated with the selective pressure of ADT and include AR gene overexpression, AR gene mutation, AR splice variants expression, and upregulation of transcriptional coactivators^[24]^.

## Mechanisms of resistance to new generation hormonal therapies

Despite the significant benefits of NHA, some CRPC patients do not respond to therapy because of primary resistance. In addition, all patients treated will acquire resistance after a certain period from the beginning of treatment. Primary resistance is commonly defined as treatment failure within the first 3 months after commencing treatment, due to clinical progression, with or without radiological confirmation^[25]^. According to this accepted definition of primary resistance, acquired resistance is considered as treatment failure occurring later during treatment. A significant proportion of patients treated with NHA develop primary resistance^[26]^, and consequently an understanding of the mechanisms of resistance is essential in order to develop new therapeutic strategies.

There are few data regarding the mechanisms of resistance that occur during treatment with apalutamide. In the pivotal SPARTAN trial, the frequency of AR signalling anomalies detected in the apalutamide group at the end of treatment was low, and AR aberrations were associated with a shorter median progression-free survival only in the placebo group^[27]^. In a recent preclinical study, apalutamide and enzalutamide exhibited agonist activity in resistant PC cell lines due to a missense mutation (F876L) in the AR LBD. The AR F876L mutation was detected in plasma samples collected from patients with progressive CRPC after apalutamide treatment. Naïve samples lacked the mutation, suggesting that F876L is involved in the development of resistance to second generation antiandrogens^[28]^. No data are currently available about the mechanisms of resistance to darolutamide.

Adaptive resistance of PC to abiraterone and enzalutamide treatment is much better understood, and it can be due to the activation of both dependent and independent AR signalling pathways^[26,29]^.

## A brief overview on AR-dependent pathways

AR amplification/overexpression is the most common genomic aberration in CRPC patients; up to 80% of these patients show AR overexpression^[30]^. This type of adaptation is more common in patients who progressed during new generation hormonal therapy than in treatment-naïve patients, so it has been considered as a potential resistance mechanism^[31]^.

AR mutations can be found in up to 30% of CRPC patients treated with ADT. Their incidence may increase during treatment with abiraterone and enzalutamide because, when AR signalling is more effective suppressed, clonal selection of tumour cells can enhance AR somatic mutations and the consequent aberrant transcription. Most AR point mutations are clustered in the LBD, altering the steroid-binding pocket and enabling its activation by alternative ligands including progesterone, hydrocortisone, oestradiol, and some AR antagonists^[32]^.

Transcriptionally activated AR splice variants (ARVs) play a critical role in the development and progression of CRPC. ARVs lack the LBD, remaining constitutively active even without androgen binding^[33]^. ARV expression is significantly increased during ADT and is related to PC progression. The most common AR splicing variant, AR-V7, is associated with resistance to both abiraterone and enzalutamide and with short survival^[34]^.

Intra-prostatic synthesis can be an important source of androgens under the selective pressure of hormonal treatment. The intra-tumour synthesis of steroid hormones, usually limited in naïve primary PC, significantly increases in CRPC patients^[35]^. Studies in CRPC xenografts have shown that several genes involved in the androgen synthesis pathway, including CYP17A1, are upregulated during hormonal therapy^[36]^. The raised intra-tumoural level of androgens stimulates both paracrine and autocrine activation of AR, irrespective of serum androgens levels^[37]^.

## A focus on AR-independent pathways

### Overexpression of the glucocorticoid receptor and progesterone receptor

The glucocorticoid receptor (GR) is a component of the steroid nuclear receptor family. Similarly to the AR, it is composed of three functional modules: the N-terminal binding, the DNA binding and the ligand binding domain. Acquired resistance to NHA can occur due to an increased expression of the GR, that shares response elements with the AR in various target genes. Puhr *et al.*^[38]^ examined GR expression and function in cell lines and human tissues of PC patients. They observed that the GR is expressed minimally in primary PC tissue and that GR expression notably increases during long-term treatment with enzalutamide. These findings confirm previous results of a study by Arora *et al.*^[39]^ that showed how GR overexpression confers clinical resistance to enzalutamide. In particular, AR inhibition resulted in GR upregulation due to the lack of AR-mediated feedback repression on GR expression. The GR substituted for the AR to activate AR target genes crucial for the maintenance of a resistant state. In the same study, the GR agonist dexamethasone was shown to induce in vitro resistance to enzalutamide while a GR antagonist restored sensitivity. The frequency and the clinical relevance of GR-driven CRPC are currently not known.

The progesterone receptor (PR) also belongs to the steroid nuclear receptor family and has been shown to be increased in CRPC. PR isoforms A and B are expressed in prostate stromal fibroblasts and smooth muscle cells, but not in epithelial cells. Reciprocal interactions between epithelium and stroma are crucial for the maintenance of prostate homeostasis and functioning. Both PR isoforms are known to regulate cell proliferation^[40]^. The PR can be involved in resistance to NHA through continued progesterone production. These findings prove a mechanism of escape from AR blockade via alternative nuclear receptors during drug exposure. Therapeutic inhibition of GR activity by mifepristone, a steroidal GR and PR antagonist, prevented CRPC growth and delayed progression in preclinical models^[41]^. A phase II study evaluating enzalutamide in addition to mifepristone in patients with mCRPC is ongoing to determine if the combination of the two drugs delays time to PSA progression (NCT02012296, Table 2). In a phase I/II study, Jayaram *et al.*^[42]^ showed that PR inhibition by onapristone, in patients with CRPC progressing after abiraterone or enzalutamide, was feasible and safe. In a prospectively defined exploratory analysis, they reported a significantly longer radiographic PFS in patients with a normal plasma AR status.

**Table 2:** Ongoing clinical trials evaluating new strategies to overcome AR-independent mechanism of resistance to new generation hormonal therapy

Mechanism of resistance	NCT	Intervention/Treatment	Status*
GR/PR overexpression	NCT02012296	Mifepristone + Enzalutamide	Recruiting
EMT	NCT02452008	Galunisertib + Enzalutamide	Recruiting
	NCT02339168	Metformin Hydrochloride + Enzalutamide	Active, not recruiting
	NCT02640534	Metformin + Enzalutamide	Recruiting
Immune evasion	NCT02861573	Pembrolizumab + Enzalutamide, Abiraterone, Docetaxel or Olaparib	Recruiting
	NCT02787005	Pembrolizumab +/- Enzalutamide	Active, not recruiting
	NCT03338790	Nivolumab + Rucaparib, Docetaxel or Enzalutamide	Recruiting
	NCT04100018	Nivolumab + Docetaxel	Recruiting
	NCT03016312	Atezolizumab + Enzalutamide	Active, not recruiting
	NCT03177187	AZD5069 + Enzalutamide	Recruiting
PI3K/AKT/mTOR pathway	NCT02125084	Everolimus + Enzalutamide	Active, not recruiting
	NCT02833883	CC-115 + Enzalutamide	Active, not recruiting
	NCT02407054	LY3023414 + Enzalutamide	Completed
	NCT02106507	Everolimus + Apalutamide	Active, not recruiting
	NCT03072238	Ipatasertib + Abiraterone	Active, not recruiting
HGF/MET pathway	NCT02207504	Crizotinib + Enzalutamide	Active, not recruiting

*Updated at June 2020. AR: androgen receptor; GR: glucocorticoid receptor; PR: progesterone receptor; EMT: epithelial-mesenchymal transition

### Epithelial-mesenchymal transition

The transition from epithelial into mesenchymal cells is a morphological phenomenon that includes the interruption of cell polarity, the acquisition of mesenchymal phenotype, and the reorganization of the cytoskeleton. The epithelial-mesenchymal transition (EMT) process is crucial for physiological embryonic development and differentiation of the urinary genital system, but it is also activated in pathological conditions, such as fibrosis or cancer progression^[43]^. While the epithelial phenotype is characterized by apical–basal cell polarity and strong intercellular adhesions, mesenchymal cells are totally different, being neither adherent nor polarized^[44]^. EMT can be induced by ADT in metastatic PC and it confers invasive potential to tumour epithelial cells. Snail and Twist are transcription factors able to induce the repression of E-cadherin expression, a key event in EMT^[45]^. The TGF-β superfamily members induce Snail transcriptional factor. Aberrant TGF-β pathway and the protein kinase C (PKC)/Twist1 signalling activation are putative mechanisms for the occurrence of resistance. There are currently no direct Twist inhibitors available, but Shiota *et al.*^[46]^ investigated a PKC inhibitor to switch back resistance in PC cell lines with promising results. A phase II trial (NCT02452008, Table 2) is ongoing to test the efficacy of the TGF-β inhibitor galunisertib in combination with enzalutamide in mCRPC. Therapeutic targeting of EMT by a proteasome inhibitor suppressing Snail seems to be promising in mCRPC^[47]^.

Recently, Liu *et al.*^[48]^ demonstrated that metformin, an oral hypoglycaemic agent, is able to revert enzalutamide resistance restoring sensitivity to the drug in mice xenografts by the inhibition of EMT. Based on the effect of metformin on the activation of STAT3 and expression of TGF-β1, the authors proposed that metformin exerts its anticancer effect by targeting the TGF-β1/STAT3 axis, involved in EMT activation. Currently, there are two ongoing clinical trials evaluating the anticancer role of metformin in combination with enzalutamide in mCRPC (NCT02339168 and NCT02640534, Table 2). Unexpectedly, a swiss single-arm phase II study showed that the addition of metformin to abiraterone is not beneficial in patients with mCRPC and PSA progression. In addition, despite the good tolerance of metformin, the authors observed a higher-than-expected gastrointestinal toxicity, due to the association of the two drugs^[49]^.

### Neuroendocrine transformation

A subset of patients with advanced PC may eventually develop a neuroendocrine (NE) phenotype as an adaptive response to intense AR signalling inhibition. An aggressive NE phenotype can also appear de novo, but it more commonly arises during hormonal therapy. The prevalence of neuroendocrine prostate cancer (NEPC) is approximately 1% in primary PC and up to 25%-30% in mCRPC^[50]^. The NE phenotype is characterized by rapid progression of disease with a low or only moderate increase in prostate specific antigen (PSA) levels. These patients are usually treated with cisplatin or carboplatin combined with etoposide, obtaining high response rates but short duration of disease control^[51]^. A systematic review of published clinical cases revealed a median time to development of NEPC of 20 months and a median OS after NEPC diagnosis of 7 months^[52]^.

**Figure 1:**
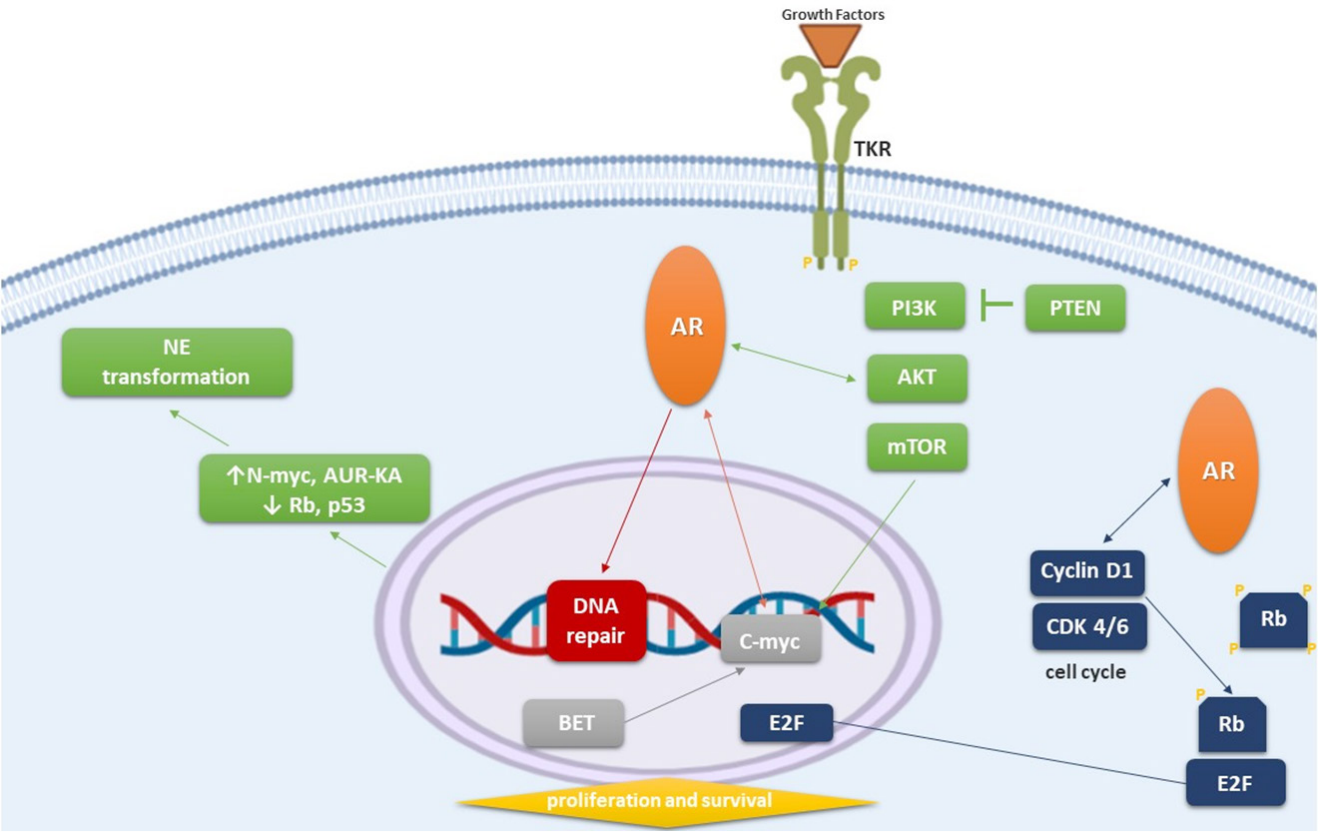
AR-independent mechanisms of resistance to new generation hormonal therapy and interaction with AR signalling. AR: androgen receptor; NE: neuroendocrine transformation; TKR: tyrosine kinase receptor; CDK: cyclin dependent kinase

NEPC is histologically different from PC, and is characterized by small round blue cells, which do not express AR or secrete PSA, but express NE markers such as chromogranin A and neuron specific enolase. Recent genomic profiling studies have shown a significant overexpression and gene amplification of Aurora Kinase A (AUR-KA) and N-myc in 40% of NEPC and 5% of advanced PC, and a loss of AR target gene expression^[53]^. AUR-KA is best known for its role in mitosis, but its interactions with the oncogene N-myc are not completely understood. In neuroblastoma, a positive feedback loop has been described in which AUR-KA induces and stabilizes N-myc^[54]^ [Figure 1]. A phase II trial was recently conducted to evaluate if AUR-KA could be a new potential target in the treatment of NEPC. 60 patients were treated with alisertib, an oral AUR-KA inhibitor. Although the study did not meet its primary endpoint of 6-month radiographic progression free survival, a subset of patients with molecular features supporting AUR-KA and N-myc activation achieved a significant clinical benefit. In particular, two patients achieved a remarkable response, with complete regression of liver lesions^[55]^. Alisertib was also studied in combination with abiraterone in patients with mCRPC progressing during abiraterone treatment. This phase I/II trial was stopped early because of intolerable toxicity and no clear signs of therapeutic activity^[56]^.

Recent studies have shown that enzalutamide treatment may cause a lower expression of the repressor element 1 silencing transcription factor (REST), a mediator of AR activity that plays a leading role in restricting neuronal differentiation of stem cells. Preclinical tests revealed that in LNCaP cells subjected to prolonged incubation with androgen-depleted media, the reduced expression of REST coincided with an increased expression of chromogranin A. Gene expression profiling demonstrated that REST not only acts to repress neuronal genes but also genes involved in cell cycle progression, including AUR-KA^[57]^. The demonstration that REST plays a role in the prostate epithelium differentiation, and that its expression is negatively related to the recurrence of disease, suggests a possible role of REST as a prognostic biomarker.

Preclinical studies have shown that aggressive variants of PC are characterized by combined alterations in RB1, Tp53 and/or PTEN^[58]^. The retinoblastoma tumour suppressor gene 1 (RB1) is a key factor in cell cycle control. RB1 is more commonly mutated in metastatic- and recurrent-PC than in primary tumours, particularly in NEPC variants^[59]^. This indicates that there is a selective pressure for RB1 loss which facilitates lineage plasticity and progression of PC cells initiated by PTEN mutation. The additional loss of the tumour suppressor gene Tp53 causes resistance to antiandrogen therapy [Figure 1]. The correlation between RB1 activity and AR-regulated genes is well known; when RB1 expression decreases, the AR signalling increases^[60]^. Cycline-D kinase inhibitors, such as palbociclib, ribociclib, and abemaciclib, may prevent RB1 phosphorylation and inactivation, restoring sensitivity to hormonal treatment. Gene expression profiling studies revealed that both mouse and human NEPC exhibit increased expression of epigenetic reprogramming factors such as the enhancer of zeste homolog 2 (Ezh2)^[61]^. Ezh2 inhibitors, including GSK-126/343/503, have recently been developed and they demonstrated restoration of AR expression and sensitivity to antiandrogen therapy in PTEN and RB1 double-knockout mice^[61]^.

### Immune evasion

Immune escape strategies protect PC from detection and destruction by the immune system. In physiological conditions, immune checkpoint pathways are essential for self-tolerance preservation and modulation of normal immune responses. Increased expression of immune checkpoint molecules by tumour cells deeply affects specific T-cell immunity in the tumour microenvironment. This mechanism inhibits the elimination of cancer cells, therefore facilitating tumour progression^[62]^. The immune checkpoints CTLA4 and PD-1/PD-L1 are the main targets of immunotherapy. They have been shown to negatively modulate cytotoxic T lymphocyte activity in tumours, enabling immunological escape^[63]^. In contrast to several other tumours, advanced PC is characterized by minimal immune cell infiltrate and a relatively low mutational burden, suggesting a possible lower sensitivity to treatment with immune checkpoint inhibitors (ICIs)^[64]^. However, mCRPC showed higher mutational load compared to primary tumors^[65]^.

Bishop *et al.*^[66]^ demonstrated that patients progressing during enzalutamide treatment had significantly increased PD-L1/2+ dendritic cells and a high frequency of PD-1+ T cells in blood, compared to those naïve or responding to treatment.

Pembrolizumab, an anti-PD-1 antibody, showed preliminary antitumor activity in PD-L1 positive mCRPC patients in the phase Ib KEYNOTE-028 trial^[67]^. In a phase II study, pembrolizumab in monotherapy was tested in 258 docetaxel-refractory patients already treated with at least one new generation hormonal therapy. A complete or partial response was observed in about 5% of patients, irrespective of their PD-L1 status. The median duration of response was not reached in PD-L1 positive disease^[68]^.

The use of PD-1 and PD-L1 as predictive biomarkers of response to immunotherapy is still undefined.

A phase II single-arm study reported ICIs anti-tumour activity in mCRPC patients progressing during enzalutamide. Ten patients were treated with pembrolizumab in addition to standard dose of enzalutamide. Three subjects experienced a rapid PSA reduction and two of them achieved a partial response, including a patient with microsatellite instability, which has proven to be a predictive factor for this therapeutic approach^[69]^.

The therapeutic association of pembrolizumab with enzalutamide in mCRPC is currently being studied in the phase I KEYNOTE-365 trial (NCT02861573, Table 2) and in the phase II KEYNOTE-199 trial (NCT02787005, Table 2).

The CA184-043 phase III study, testing the anti-CTLA4 ipilimumab vs placebo after RT in mCRPC patients progressing after chemotherapy with docetaxel, did not reach statistical significance for its primary endpoint of OS. Despite this, a possible benefit in patients with a favourable prognostic profile has been reported in a post hoc analysis^[70]^.

Monotherapy with nivolumab, another anti-PD-1 antibody, did not show any activity in two phase I trials enrolling pre-treated mCRPC patients^[71,72]^. Nivolumab is being studied in a phase II trial in combination with either rucaparib, docetaxel, or enzalutamide in mCRPC patients (NCT03338790, Table 2), and in a phase III trial in association with docetaxel in mCRPC chemo-naïve patients who have progressed after second-generation hormonal therapy (NCT04100018, Table 2).

Efficacy data of atezolizumab are still in the early stages^[73]^. The phase III IMbassador250 study is testing atezolizumab in addition to enzalutamide in patients with mCRPC who are ineligible to taxane chemotherapy after failure of androgen synthesis inhibitor treatment (NCT03016312, Table 2).

Ongoing clinical trials are examining new agents, such as AZD5069, a chemokine receptor antagonist, in association with enzalutamide in mCRPC (NCT03177187, Table 2).

Currently, the only FDA-approved immunotherapy in mCRPC patients with asymptomatic or minimally symptomatic mCRPC is sipuleucel-T, an autologous vaccine that stimulates T cell immune responses against the tumour^[74]^.

Although the early data regarding ICIs are promising, their use in clinical practice for PC is still quite limited.

## Activation of other pathways

### The HER2/HER3 pathway

Anomalies in the HER2/HER3 pathway, which lead to the activation of PI3K/AKT signalling, may represent a targetable mechanism of resistance to NHA. The cross talk between the HER2/HER3 and AR has been extensively described. HER2 expression is induced by enzalutamide treatment through AR activity modulation. HER2-dependent AR activation has shown to be inhibited in vitro by treatment with the EGFR/HER2 inhibitor lapatinib, able to reduce cell viability and increase apoptosis. Combination treatment with lapatinib with enzalutamide improved the response rate compared to enzalutamide alone in both *in vitro* and *in vivo* models^[75]^. Similarly, Gao *et al.*^[76]^ demonstrated that ErbB2 signalling was enhanced and associated with AR re-activation in abiraterone-resistant CRPC xenograft models. Furthermore, concomitant treatment with abiraterone and lapatinib in CRPC cell lines blocks AR reactivation and suppresses tumour progression.

## The PI3K-AKT-mTOR pathway

The PI3K-AKT-mTOR pathway plays a key role in cell cycle regulation and modulates all the principal cellular processes such as cell growth, proliferation, apoptosis and protein synthesis. Its aberrant activation is involved in PC development and progression^[77]^ [Figure 1]. The PI3K/AKT/mTOR pathway has been found altered in 20%-40% of primary PC and in nearly 50% of mCRPC^[78]^.

Carver *et al.*^[79]^ demonstrated the cross talk between the AR and PI3K in murine models and human xenografts. The inhibition of one pathway activates the other; PI3K pathway inhibition activates AR signalling by relieving the feedback inhibition of HER kinases. Likewise, AR inhibition activates AKT signalling by reducing levels of the AKT phosphatase PHLPP (PH domain leucine-rich repeat protein phosphatase). Synergistic inhibition of PI3K and AR signalling induced PC regression, indicating that both pathways coordinate to support cell survival^[79]^. Thomas *et al.*^[80]^ confirmed these results both *in vitro* and *in vivo*, examining the association of the PI3K/AKT inhibitor capivasertib with the antiandrogen bicalutamide. The combined targeting of PI3K/AKT pathway and AR axis significantly delayed CRPC progression. Recently, results of a phase I dose-escalation study of enzalutamide in combination with capivasertib were published, showing anti-tumour activity of the combination therapy in the subgroup of patients harbouring aberrations in the PI3K/AKT/mTOR pathway^[81]^. The PI3K isoform p110β regulates cell mitosis and survival. BL140, a newly developed isoform β-specific inhibitor, has been shown to effectively suppress PC growth in all PC lines tested^[82]^.

Another potential therapeutic strategy under investigation is the direct inhibition of mTOR. A phase II study showed a limited clinical efficacy of single-agent mammalian target of rapamycin (mTOR) inhibitor in mCRPC, due to dose reductions secondary to toxicity^[83]^. Two phase I trials are testing a combination strategy of enzalutamide with the mTOR inhibitors everolimus (NCT02125084, Table 2), or CC-115 (NCT02833883, Table 2), in mCRPC. At the 2019 American Society of Clinical Oncology annual meeting, Sweeney and colleagues presented results of a randomized Phase Ib/II study of the PI3K/mTOR dual inhibitor LY3023414 with or without enzalutamide in patients with mCRPC progressing despite abiraterone treatment (NCT02407054, Table 2). With a manageable safety profile, the combination of LY3023414 and enzalutamide met the primary endpoint of PSA-PFS, which was supported by the exploratory finding of a clinically meaningful improvement in rPFS in AR-V7 negative patients^[84]^. A phase I study of abiraterone acetate combined with dactolisib, a dual PI3K/mTOR inhibitor, was closed early because the 50% of patients experienced dose-limiting toxicity^[85]^. The efficacy and safety of apalutamide plus everolimus in mCRPC patients progressing after treatment with abiraterone acetate is under investigation in a phase I trial (NCT02106507, Table 2).

Studies with AKT inhibitors have shown some encouraging results. In a phase II study, ipatasertib in addition to abiraterone demonstrated superior anti-tumour activity compared to abiraterone alone in docetaxel-pre-treated mCRPC patients, particularly in those with loss of PTEN, a negative regulator of PI3K signaling^[86]^. These findings support PTEN loss as a predictive factor for treatment response. A phase III trial is testing the addition of ipatasertib to abiraterone in men with asymptomatic or mildly symptomatic, previously untreated mCRPC (NCT03072238, Table 2).

### Autophagy

Autophagy is a catabolic process of self-digestion in which cellular components are isolated from the rest of the cell within autolysosomes for degradation and recycling, in order to maintain cellular homeostasis. The physiological balance between autophagy and apoptosis can be lost in cancer development because of a dysfunction in apoptosis. The subsequent upregulation of autophagy increases nourishment for high-proliferative tumour cells^[87]^. There are different kinds of autophagy: macro-, micro- and chaperone-mediated autophagy. Macro-autophagy is related to drug resistance in several types of cancer and it represents an adaptive response to sustain cell survival under metabolic stress, such as androgen deprivation^[88]^. The AR acts as a key modulator of autophagy. Treatment with bicalutamide on PC cell lines has shown to induce autophagy^[89]^. Nguyen *et al.*^[90]^ reported a similar effect with enzalutamide treatment in CRPC cell lines. Enzalutamide induced autophagy by activation of AMP-dependent protein kinase (AMPK) and suppression of mTOR. In addition, the authors demonstrated that small interfering RNA targeting AMPK significantly repressed autophagy and increased cell death in PC cells exposed to enzalutamide or conventional ADT, suggesting that autophagy represents a crucial survival mechanism in CRPC. Lastly, modulators of autophagy such as metformin and clomipramine significantly enhanced enzalutamide activity and reduced cancer growth in mouse models implanted with enzalutamide-resistant cells.

Resistance to hormonal therapy is often associated with stress-induced activation of molecular chaperones involved in the autophagy process. Clusterin (CLU) is a heat-shock chaperone-like protein induced by antiandrogens such as enzalutamide. CLU inhibition by OGX-011 repressed enzalutamide-induced activation of AKT and MAPK pathways. The inhibition of both AR (with enzalutamide) and CLU (with OGX-011) synergistically increased apoptotic rates and delayed the appearance of CRPC in LNCaP tumour and PSA progression in vivo^[91]^.

### The C-myc pathway

The oncogene C-myc promotes cells growth and proliferation by increasing ribosome biosynthesis. Bromodomain and extra-terminal enhancer (BET) proteins are involved in regulating chromatin remodelling by C-myc [Figure 1]. In the normal prostate, C-myc is negatively regulated by AR-mediated signalling. The C-myc oncogene is frequently overexpressed in mCRPC without NE features and plays a significant role in driving PC tumour genesis. AR-signalling-competent human CRPC cell lines have demonstrated sensitivity to BET inhibition. *In vivo*, BET inhibitors were more effective than direct AR antagonists in blocking cell proliferation in mouse models with CRPC xenografts^[92]^. In enzalutamide-resistant PC models, enzalutamide and apalutamide showed stronger tumour growth inhibition when associated with BET inhibitors, JQ1 and OTX015, respectively^[93]^.

### The HGF/MET pathway

MET is a tyrosine kinase receptor activated by a unique ligand, known as hepatocyte growth factor (HGF). The HGF/MET signalling system plays a fundamental role in tumour growth, invasion, and in metastasis development in many types of malignancies. MET overexpression has been described in primary and advanced PC and it correlates with poor prognosis^[94]^.

Verras *et al.*^[95]^ showed that the AR represses c-MET expression in a ligand-dependent manner in PC xenografts. Although ADT can restrain the expression of growth-promoting genes activated by the AR, it may also reduce the repressive role of androgen signalling on c-MET expression. Accordingly, there is a strong rationale supporting the combination of inhibition of the HGF/MET pathway with ADT.

Cabozantinib is an orally available receptor tyrosine kinase inhibitor with strong activity against MET and VEGFR2. The safety profile of cabozantinib in combination with abiraterone was defined in a phase I study^[96]^. The phase II trial testing the same combination was stopped early after the results of the phase III study COMET1, in which cabozantinib did not significantly improve OS compared to prednisone in heavily treated mCRPC patients^[97]^.

Preclinical work has revealed that crizotinib, a multi-kinase inhibitor including MET, can repress PC growth in cell lines and mouse models. A phase I trial is ongoing to study the toxicity and pharmacokinetic profile of crizotinib in association with enzalutamide in mCRPC, before or after treatment with docetaxel (NCT02207504, Table 2).

### The NF-κB/p52 pathway

The activation of the NF-κB/p52 pathway plays a role in the development of resistance to enzalutamide. Nadiminty *et al.*^[98]^ demonstrated activation of NF-κB/p52 and enhanced expression of ARV7 in PC cell lines subjected to long-duration treatment with enzalutamide. In contrast, in vitro downregulation of NF-κB/p52 reduced ARV7 expression and re-sensitized PC cells to enzalutamide. NF-κB normally regulates the expression of different cytokines. Interleukin-6 (IL-6) is highly expressed in CRPC and it regulates the transcriptional activity of the AR. Experimental drugs targeting IL-6 showed unsuccessful results, however.

### The DNA repair pathway

Human DNA is constantly exposed to damage by endogenous and exogenous agents, and a complex repair system is active to protect cells’ genome stability. Poly-ADP-ribose polymerase (PARP) enzymes are primarily involved in detecting single strand damage and activating the repair process [Figure 1]. Aberrations in key genes of DNA repair pathways, including somatic and germline mutations in BRCA2, or biallelic loss of ATM, were found in 19% of primary PC and almost 23% of mCRPC^[81]^. The identification of defects in the DNA repair mechanism provides a strong rationale for the use of PARP inhibitors (PARPi) in PC treatment. The phase II TOPARP-A study demonstrated that olaparib treatment in patients who did not benefit from standard therapies, and who had defects in DNA-repair genes, was associated with high response rates^[99]^. In 2016 the FDA designated olaparib as an innovative therapy for mCRPC patients with BRCA1/2 or ATM gene mutation who have received prior chemotherapy with a taxane and at least one new generation hormonal therapy. PARP1 is also implicated in the regulation of AR transcriptional activity. The AR requires active PARP1 to modulate its association with chromatin. There is accumulating evidence that second-generation AR signalling inhibitors might induce “BRCAness”^[100-102]^ and there is potential synergy between AR inhibitors and PARP inhibitors in the treatment of mCRPC^[103]^. Proof of the synergy between PARPi and hormonal agents derives from a randomized phase II trial testing the combination of olaparib with abiraterone in docetaxel-pretreated mCRPC patients. A benefit in rPFS as primary endpoint was shown in the experimental group, regardless of DNA repair genes mutation status^[104]^. Combination therapy with NHA and PARPi is also being studied in the first-line setting of mCRPC. Pre-clinical studies suggested that the combination of enzalutamide and PARPi can lead to BRCAness because of the ability of enzalutamide to down-regulate the expression of genes involved in the DNA repair system. The rationale of testing this co-treatment as first-line therapy is to induce the BRCAness phenotype and to expand the use of PARPi in the treatment landscape of mCRPC. Given the cross-talk between the AR and the DNA repair system, several studies are ongoing to test the activity of PARPi in combination with new generation hormonal therapies in mCRPC.

## Conclusion

The advent of new generation hormonal therapies has given way to a new era in the treatment of CRPC. Second generation antiandrogens have increased specificity and higher affinity for the AR than previous hormonal agents, resulting in more effective suppression of AR signalling. These drugs significantly improve patients’ quality of life and prolonged overall and metastasis-free survival. Despite these advances, a proportion of patients do not benefit at all from the treatment and, among those who respond, resistance will undoubtedly occur after a variable time. Primary and acquired resistance is a multifactorial phenomenon that is caused not only by AR-dependent mechanisms, but also by the activation of other pathways bypassing AR signalling. Metastatic PC is currently understood to be a heterogeneous disease, characterized by a coexistence of AR-driven and AR-independent neoplastic cells. This heterogeneity is the prime driver for distinct biological behaviours observed and for the different responses to NHA. In recent years, many efforts have been made to investigate the genomic landscape of PC. The aim is to change the future of healthcare by moving towards precision medicine and finding the best therapeutic approach for individual patients. Genomic sequencing of PC patient samples led to the discovery of potentially targetable gene mutations, such as PI3K/AKT, MET, BRCA2, ATM, and others. Several novel agents targeting the identified non-AR-driven pathways involved in the pathogenesis and progression of PC are currently under investigation to prevent or overcome these mechanisms of resistance, some with very promising results. PARP inhibitors are the most advanced molecules in clinical trials for CRPC, both as single agents or in combination with hormonal therapy. The future challenge for clinicians will be to better understand the biological heterogeneity of PC and to select patients with a high chance of response to different treatments. *In vitro* and *in vivo* studies are ongoing with the aim of detecting predictive biomarkers of response, and to investigate new approaches to bypassing resistance.

## Declarations

### Copyright

© The Author(s) 2020.
